# Chemical Characteristics and Anticancer Activity of Essential Oil from *Arnica Montana* L. Rhizomes and Roots

**DOI:** 10.3390/molecules25061284

**Published:** 2020-03-12

**Authors:** Piotr Sugier, Joanna Jakubowicz-Gil, Danuta Sugier, Radosław Kowalski, Urszula Gawlik-Dziki, Barbara Kołodziej, Dariusz Dziki

**Affiliations:** 1 Department of Botany, Mycology and Ecology, Institute of Biological Sciences, Maria Curie-Skłodowska University, 19 Akademicka Street, 20-033 Lublin, Poland; piotr.sugier@poczta.umcs.lublin.pl; 2 Department of Functional Anatomy and Cytobiology, Institute of Biological Sciences, Maria Curie-Skłodowska University, 19 Akademicka Street, 20-033 Lublin, Poland; jjgil@poczta.umcs.lublin.pl; 3 Department of Industrial and Medicinal Plants, University of Life Sciences in Lublin, 15 Akademicka Street, 20-950 Lublin, Poland; danuta.sugier@up.lublin.pl (D.S.); barbara.kolodziej@up.lublin.pl (B.K.); 4 Department of Analysis and Evaluation of Food Quality, University of Life Sciences in Lublin, 8 Skromna Street, 20-704 Lublin, Poland; radoslaw.kowalski@up.lublin.pl; 5 Department of Biochemistry and Food Chemistry, University of Life Sciences, Skromna 8, 20-704 Lublin, Poland; 6 Department of Thermal Technology and Food Process Engineering, University of Life Sciences, Głęboka 31, 20-612 Lublin, Poland; dariusz.dziki@up.lublin.pl

**Keywords:** *Arnica montana* L., rhizomes, roots, essential oil, anticancer activity, T98G and MOGGCCM cell lines

## Abstract

*Arnica montana* L. is a medicinal plant with diverse biological activities commonly used in pharmacy and cosmetics. The attributes of *A. montana* are mainly related to the concentration and chemical composition of essential oils (EOs). Therefore, the objective of this study was to characterize the chemical composition of EOs derived from *A. montana* rhizomes and roots taking into account the age of the plants and to investigate the effect of the analyzed EOs on induction of apoptosis, necrosis, and autophagy in human glioblastoma multiforme T98G and anaplastic astrocytoma MOGGCCM cell lines. Rhizomes and roots of mountain arnica were harvested at the end of the third and fourth vegetation periods. The chemical composition of essential oils was determined with the GC–MS technique. Among the 37 components of the essential oil of *A. montana*, 2,5-dimethoxy-p-cymene (46.47%–60.31%), 2,6-diisopropylanisole (14.48%–23.10%), thymol methyl ether (5.31%–17.79%), *p*-methoxyheptanophenone (5.07%–9.65%), and α-isocomene (0.68%–2.87%), were detected in the rhizomes and roots of the three-year-old plants and in the rhizomes and roots of the four-year-old plants. The plant part (rhizome, root) and plant age can be determinants of the essential oil composition and, consequently, their biological activity. The induction of apoptosis (but not autophagy nor necrosis) at a level of 28.5%–32.3% is a promising result, for which 2,5-dimethoxy-p-cymene, 2,6-diisopropylanisole, thymol methyl ether, and p-methoxyheptanophenone are probably mainly responsible. The present study is the first report on the anticancer activities of essential oils from *A. montana* rhizomes and roots.

## 1. Introduction

Natural products and their derivatives are increasingly desirable sources of novel therapeutic molecules worldwide [1]. Researchers’ attention is focused on the chemical characteristic of essential oils (Eos) derived from plants and the wide range of their biological properties, including antioxidant, antimicrobial, antiviral, antimutagenic, anti-inflammatory, immunomodulatory, antiprotozoal, antifungal, and anticancer activities, [2,3,4,5]. Many Asteraceae plant species have a long history of ethnopharmacological use and are important for medicinal and pharmacological purposes at present [6,7,8,9,10]. This family is represented by mountain *Arnica montana* L., i.e., an herbaceous plant species which has been used in folk medicine for many years and is a rich source of raw material abundant in secondary metabolites. Therefore, it has a number of different applications, e.g., it is widely used in pharmaceutical and cosmetic industries [11]. The demand for *Arnica* raw material has exerted pressure on naturally growing populations, i.e., by increased collection for medicinal purposes, which has led to a rapid decline in this species in Europe [12,13]. Moreover, natural populations of this rare and endangered plant species constitute resources of important genetic diversity on this continent and are a source of sesquiterpene lactones, flavonoids, terpenoids, phenolic acids, and essential oils (EOs) with antibacterial, antifungal, antiseptic, anti-inflammatory, antiradical, antisclerotic, and antioxidant activities [9,10,11,14,15,16,17,18,19,20,21,22]. Therefore, during the last decades, *Arnica* genotypes taken from natural sites and collections have been the subject of various studies on agricultural factors modifying the yield and chemical composition of raw material [9,18,21,23,24,25]. In turn, new combinations of EO components emerging under the influence of biotic and abiotic factors can exhibit new properties and, consequently, new activities that can be used in medicine, pharmacy, or cosmetic industry in the future. The age of plants tested in this study is one of the agents that modify the yield and chemical composition of EOs in medicinal plants [10,17,25].

EOs contained in the underground parts of *Arnica* have been studied for many years [20,21,22,23,24,25,26] and agricultural factors such as fertilization and histochemical localization of essential oil have recently been investigated [17,18,19,20,21,22,23,24,25,26,27]. Although *A. montana* has long been cultivated mainly for production of flower heads and many studies have been conducted to characterize the raw material [9,15,18,24], the knowledge of the chemical characteristics and biological activity of EOs from rhizomes and roots of the species is still insufficient [17]. Therefore, the results presented in this paper fill this gap and are a next step in studies of the anticancer activity of mountain *Arnica* secondary metabolites.

Currently, approximately half of conventional chemotherapy agents have plant origin, with roughly 25% directly derived from plants and 25% being chemically modified versions of phytoproducts. Natural plant components are particularly important for patients that do not tolerate extreme side effects [28]. Therefore, many alternative treatments based on plant molecules have been explored, and plants have been increasingly analyzed for their anticancer properties [28,29,30]. A report on the global burden of cancer showed that over half of ca. 18 million new cancer cases diagnosed worldwide in 2018 would cause deaths, which points to a growing global problem [31]. The major group of malignant gliomas is represented by anaplastic astrocytoma and glioblastoma multiforme. Despite the progress in conventional therapy, the prognosis for patients with gliomas remains poor, and although tremendous efforts have been made in to improve such therapies as surgery, radiotherapy, and chemotherapy, the clinical outcome of gliomas remains dismaying [29,30,31,32]. In this situation, new chemicals derived from plants are being sought, and plant EOs have become elements of treatment of various types of such malignancies as gliomas more frequently [33]. Such molecules are assumed to have potential anticancer activities useful in prevention and therapeutic strategies [34]. Therefore, there is an urgent need to search for new substances of plant origin, e.g., essential oils to elucidate the molecular basis of malignant progression of gliomas [35,36,37,38,39]. The knowledge of the impact of secondary metabolites on gliomas is insufficient. Our latest study focused on the different EO chemical profiles exhibited by plants of different age as novel sources of natural antiglioma activity [10]. However, the present study was conducted to determine a new quality of secondary metabolites in a very interesting and valuable pharmacopoeial plant species, i.e., *A. montana* [9,20,21,22]. Moreover, plant age and plant parts are other factors modifying the quality of EOs extracted from underground parts, i.e., an alternative source of biological substances. The data presented in this paper are a continuation of the search of antiglioma substances. The impact of EOs from *A. montana* rhizomes and roots on cancer cell lines is presented for the first time.

The parts of *Arnica* plants contain a wide range of secondary metabolites. Essential oils are located in flower heads, rhizomes, roots, and achenes [9,10,15,21,26,40]. The investigations of EOs contained in the flower heads of different European *A. montana* populations have shown differences in the chemical profile of EOs. The EOs obtained from plants cultivated in Poland were characterized by dominance of β-caryophyllene, caryophyllene oxide, decanal, germacrene D, and farnesyl acetate [9,21]. As reported by Ristic et al. [40], β-caryophyllene, germacrene D, and decanal dominated in the EO of *A. montana* grown on Tara Mountain in Serbia. In turn, in a study from Lithuania, Judžentienė and Būdienė [15] reported the presence of (2E, 6E)-farnesol, (2Z, 6E)-farnesyl acetate, (Z, Z)-geranyl linalool, β-caryophyllene, and caryophyllene oxide; however, the two main EO compounds were not identified. Pljevljakušić [17] demonstrated that 2,5-dimethoxy-p-cymene, thymol methyl ether, p-methoxyheptanophenone, and 2,6-diisopropylanisole were the main constituents of oils from rhizomes and roots of two-year-old and three-year-old plants growing on Tara Mountain (subalpine climate) in Serbia. Weremczuk-Jeżyna [26] showed that EOs were dominated by 10-isobutyryloxy-8,9-didehydro-thymol isobutyrate and 10-isobutyryloxy-8,9-didehydro-thymol methyl ether. In turn, Danila et al. [41] reported 35 compounds in EOs of *A. montana* rhizomes and roots collected from wild populations in the Romanian East-Carpathians, with the following major constituents: 3-t-butyl-1,2-dimethoxybenzene, thymol, tetradecane, non-3-en-2-one, and thymol methyl ether. However, there is no information on the biological activity of these oils. Recently, another new source of EOs from *Arnica montana* L. achenes and their chemical characteristics have been reported [10]. This study is the first report on the anticancer activities of essential oil from *A. montana* achenes and generally the only example of studies of the biological activity of *Arnica* EOs so far. The main components in the EOs from the *Arnica* achenes included 2,5-dimethoxy-p-cymene, cumene, thymol methyl ether, 2,6-diisopropylanisole, decanal, and 1,2,2,3-tetramethylcyclopent-3-enol. Such a wide range of different components of *Arnica* EOs implies a very broad spectrum of the possible use of these chemically different secondary metabolites.

Studies conducted lately have shown that EOs from achenes exert an anticancer effect by induction of cell death in anaplastic astrocytoma and glioblastoma multiforme cells [10]. This facilitates the use of this secondary metabolite in further studies that will contribute to development of glioma therapy in the future. Moreover, the concentration of EOs in achenes and in flower heads is similar; however, rhizomes and roots, which can contain even up to 4% of EOs in dry mass with a varied chemical composition, are a richer source of EO [17]. Therefore, the objective of this study was: (i) to characterize the chemical composition of EOs derived from *A. montana* rhizomes and roots taking into account the age of the plants, and (ii) to investigate the effect of the analyzed EOs on induction of apoptosis, necrosis, and autophagy in human glioblastoma multiforme T98G and anaplastic astrocytoma MOGGCCM cell lines.

## 2. Results

### 2.1. Chemical Characteristics of EO

**Figure 1:**
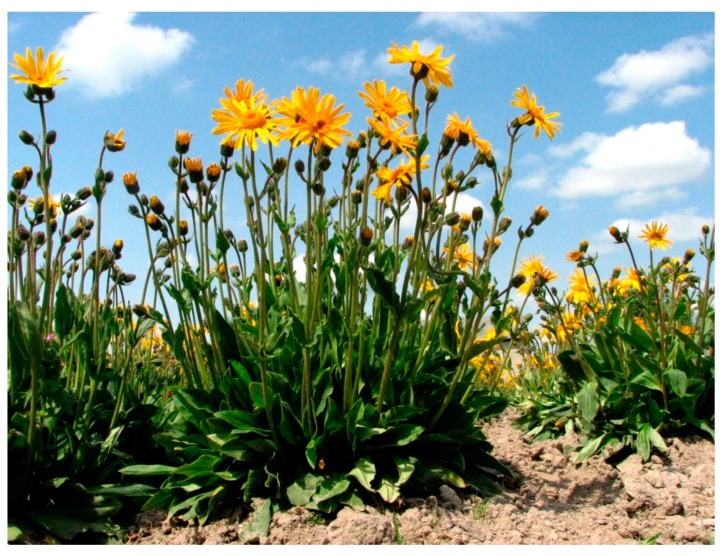
*Arnica montana* plants—3-year-old plantation.

**Figure 2:**
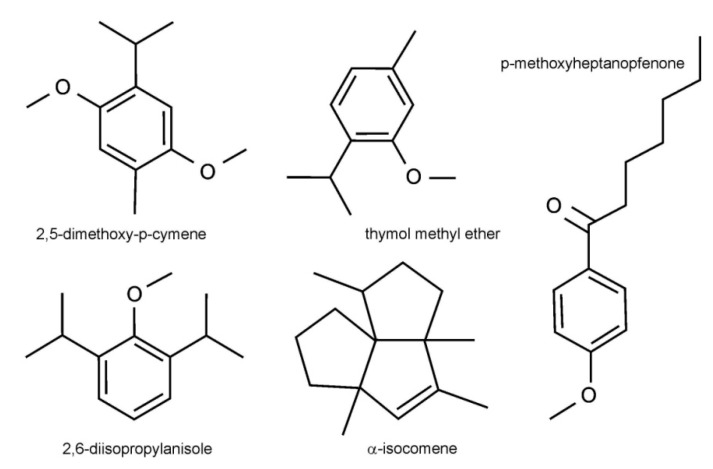
Chemical structures of selected essential oil (EO) compounds in *A. montana* rhizomes and roots.

Mountain *Arnica* plants were harvested from a three-year-old and four-year-old plantation (Figure 1). The content of the components of essential oil (EOs) obtained from the rhizomes and roots of the three-year-old (EO-RH3, EO-RO3) and four-year-old *A. montana* plants (EO-RH4, EO-RO4) is presented in Table 1. There are significant differences in the essential oil concentration between EO-RH3, EO-RO4, EO-RH3, and EO-RO4 (ANOVA; F = 105.7, *p* < 0.001). The highest average value was found in EO-RO3 (0.84%). There were slight differences in the chemical composition of EOs between the four samples. Thirty seven components in the analyzed EO-RH3, EO-RH4, EO-RO3, and EO-RO4 samples constituted 96.8%, 98.1%, 98.8%, and 98.8% of the total EOs, respectively. The studied EOs were dominated by oxygenated monoterpenes (65.61%, 67.89%, 66.67%, 65,75%) in the EO-RH3, EO-RH4, EO-RO3, and EO-RO4 samples, respectively (Table 1). Phenyls (24.13%, 24.43%, 25.97%, 27.84%) were the second major class of compounds. Another class of constituents was represented by sesquiterpenes (4.67%, 6.11%, 3.03%, 4.04%), monoterpenes (2.05%, 0.87%, 0.95%, 0.49%), and oxygenated sesquiterpenes (0.37%, 0.75%, 0.25%, 0.65%) in the EO-RH3, EO-RH4, EO-RO3, and EO-RO4 samples, respectively. The main components in the EOs from the analyzed *Arnica* were 2,5-dimethoxy-p-cymene (46.47%, 56.42%, 60.31%, 60.23%), 2,6-diisopropylanisole (14.48%, 19.78%, 17.41%, 23.10%), thymol methyl ether (17.79%, 9.18%, 5.31%, 3.87%), *p*-methoxyheptanophenone (9.65%, 6.19%, 7.02%, 5.07%), and α-isocomene (1.05%, 0.68%, 2.87%, 1.36% in EO-RH3, EO-RH4, EO-RO3, and EO-RO4, respectively (Figure 2).

### 2.2. Differentiation of the EO Content

**Figure 3:**
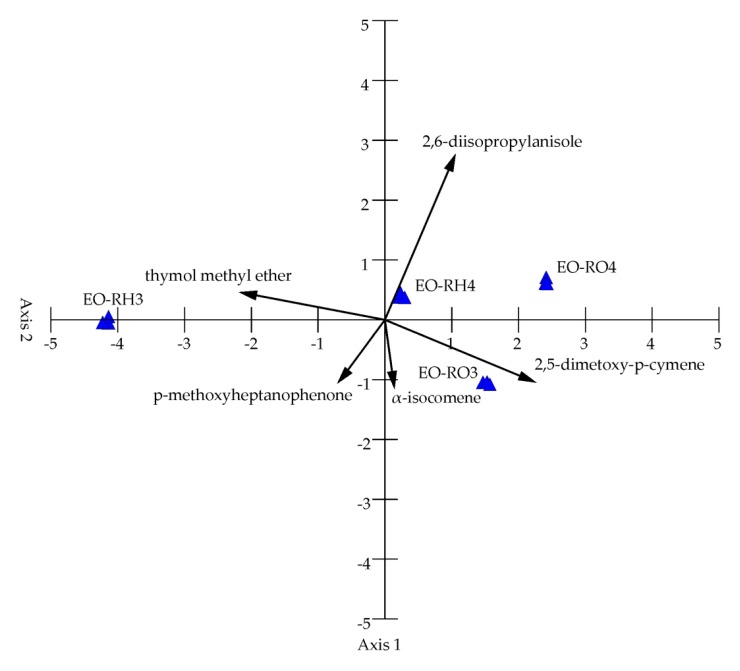
PCA diagram grouping the essential oil samples from the rhizomes and roots of the 3-year-old mountain *Arnica* plants (EO-RH3, EO-RO3) and in the rhizomes and roots of the 4-year-old plants (EO-RH4, EO-RO4).

The principal correspondence analysis (PCA) of the 37 chemical variables from the studied samples revealed two principal components with a greater effect on the chemical composition of EO, but only four main components are presented in Figure 3. The PCA analysis showed distribution of the samples along the first axis. The two PCA axes account for 98.93% of the total variance, with 92.77% of the total variance explained by the first axis; therefore, two principal components are sufficient to describe the presented samples (Table 2). Axis 1 is the linear combination of the studied oils, which better summarizes the variations in the original data matrix in a single number, whereas Axis 2 better summarizes the remaining information. The dimensionality of the data was therefore reduced from 37 variables to two uncorrelated components with 5% loss of variation.

The chemical variables are represented as a function of both Axis 1 and Axis 2. 2,5-dimethoxy-p-cymene, thymol methyl ether, 2,6-diisopropylanisole, and p-methoxyheptanophenone are the main factors determining the chemical differentiation of EOs (Figure 3, Table 2). Axis 1 shows a high positive correlation with 2,5-dimethoxy-p-cymene and a negative correlation with thymol methyl ether; therefore, this principal component separates samples with high content of 2,5-dimethoxy-p-cymene and low content of thymol methyl ether (EO-RO3, EO-RO4, EO-RH4) on the right side and samples with low content of 2,5-dimethoxy-p-cymene and high content of thymol methyl ether (EO-RH3) on the left side of the ordination space. In turn, Axis 2 of PCA shows a high positive correlation with 2,6-diisopropylanisole and a negative correlation with p-methoxyheptanophenone and α-isocomene. This principal component separates samples situated in the upper part of the ordination space with high content of 2,6-diisopropylanisole and samples located in the lower part of the ordination space with high content of p-methoxyheptanophenone and α-isocomene.

### 2.3. Anticancer Activity

**Figure 4:**
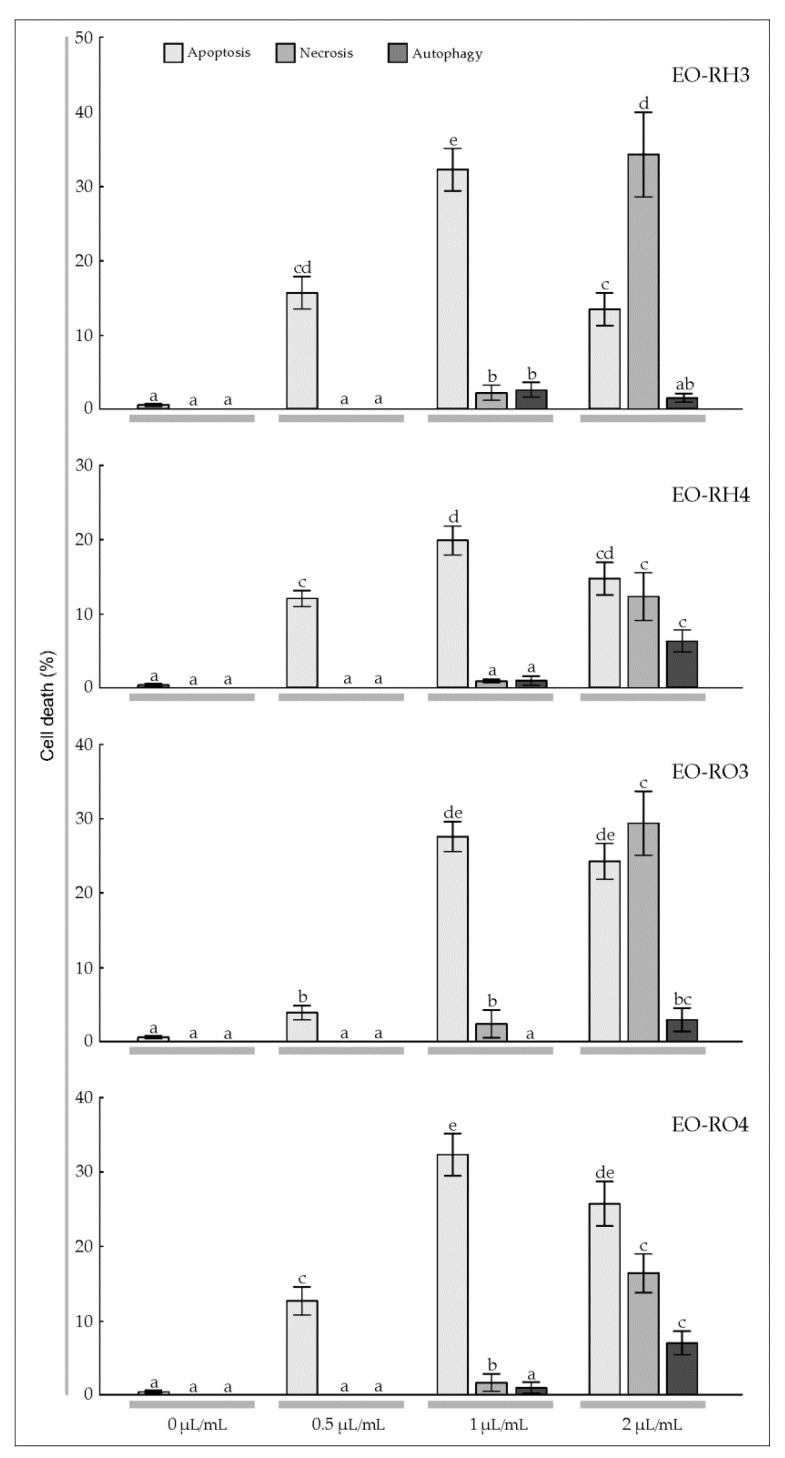
Level of apoptosis, necrosis, and autophagy induction in glioblastoma multiforme T98G cells treated with the essential oil (concentration: 0, 0.5, 1, 2 μL/mL) from the rhizomes and roots of the 3-year-old mountain *Arnica* plants (EO-RH3, EO-RO3) and the rhizomes and roots of the 4-year-old plants (EO-RH4, EO-RO4). The values designated by the different letters are significantly different (*p* = 0.05). (Tukey-test, *p* < 0.05).

**Figure 5:**
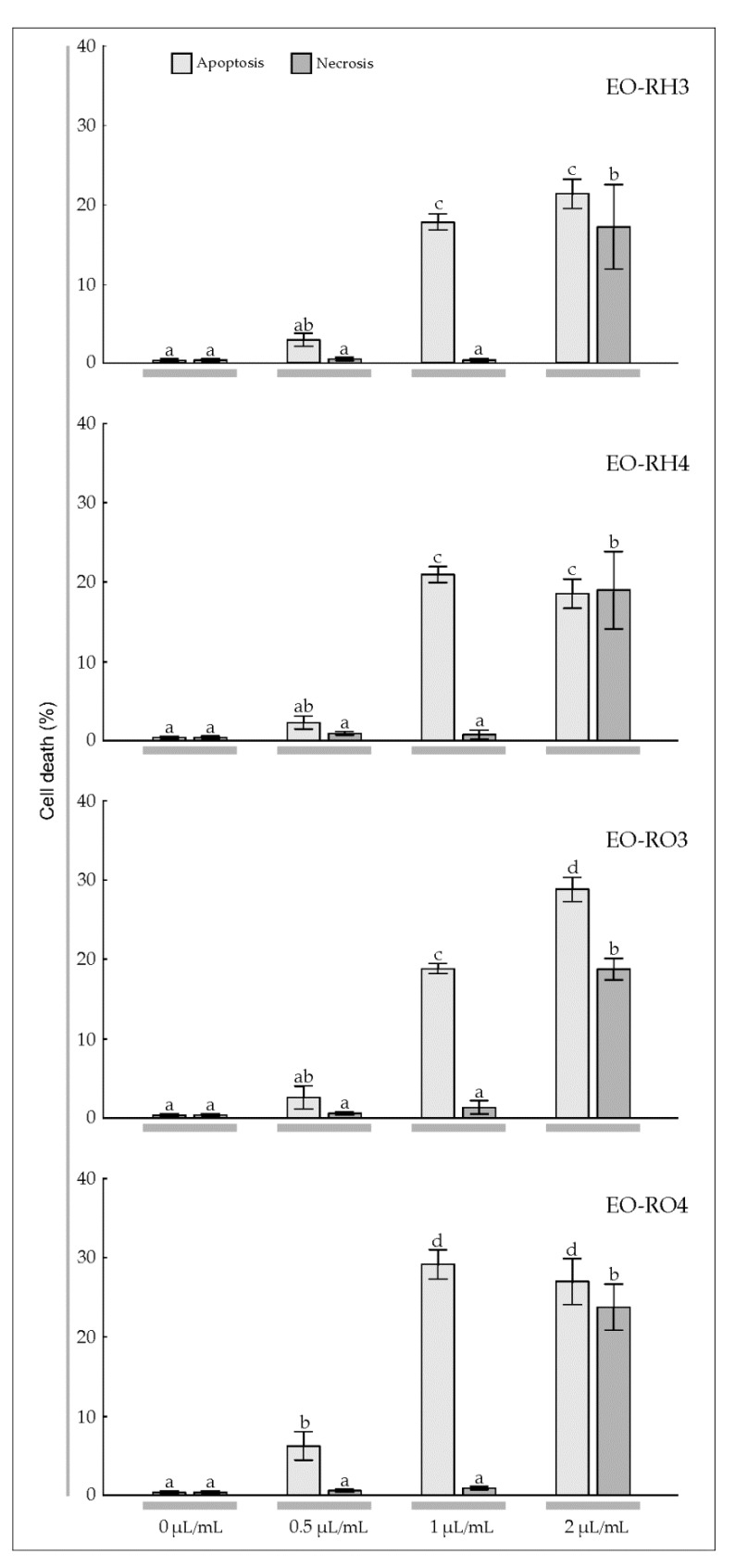
Level of apoptosis, necrosis, and autophagy induction in anaplastic astrocytoma MOGGCCM cells treated with the essential oil (concentration: 0, 0.5, 1, 2 μL/mL) from the rhizomes and roots of the 3-year-old mountain *Arnica* plants (EO-RH3, EO-RO3) and the rhizomes and roots of the 4-year-old plants (EO-RH4, EO-RO4). The values designated by the different letters are significantly different (*p* = 0.05). (Tukey-test, *p* < 0.05).

To estimate the sensitivity of T98G and MOGGCCM cells to the treatment with the EOs derived from *Arnica* rhizomes and roots, a staining method with dyes specific for apoptosis, necrosis, and autophagy; i.e., Hoechst 33342, propidium iodide, and acridine orange, respectively, was employed (Figure 4 and Figure 5). The multi-way ANOVA results showed a statistically significant main effect of the plant age (A) and the EO concentration (C); however, there was no statistically significant effect of the plant part (R) on apoptosis, necrosis, and autophagy of the T98G cells (Table 3). In the case of this cell line, there was a statistically significant effect of the R× A, R × C, A × C, and R × A × C interactions on apoptosis, a significant effect of the A × C interaction on necrosis, and a significant effect of the R × C, A × C, and R × A × C interactions on autophagy. Different results were obtained in the case of the MOGGCCM cells. The essential oils did not cause autophagy; however, all analyzed factors and their interactions were found to have a statistically significant effect on the level of apoptosis, but only the EO concentration had an effect on necrosis (Table 3).

The microscopic observations demonstrated that EO-RH3 added to the T98G culture medium had a considerable effect on induction of cell death (Figure 4, EO-RH3). A significant 15.7% increase in the number of apoptotic cells was induced by EO-RH3 at the concentration of 0.5 μL/mL (Figure 4, EO-RH3). The increase in the EO concentration to 1 μL/mL resulted in a further increase in the level of apoptosis to 32.3% and simultaneously initiated necrosis and autophagy at a level of 2.0% and 2.3%, respectively. The EO concentration of 2 μL/mL caused a decrease in the percentage of apoptotic cells (13.3%) to the level characteristic for the concentration of 0.5 μL/mL. Moreover, although apoptosis occurred at the EO concentration of 2 μL/mL, necrosis (34.7%) and autophagy (1.0%) were initiated.

The application of EO-RH4 to the T98G culture medium (Figure 4, EO-RH4) caused a similar response (Figure 4, EO-RH3). A significant 12.0% increase in the number of apoptotic cells induced by EO-RH4 was observed at the concentration of 0.5 μL/mL. A further increase in the EO-RH4 concentration to 1 μL ml also resulted in a ca. 19.7% increase in the level of apoptosis; however, this value was statistically significantly lower than the level of apoptosis caused by EO-RH3 at the concentration of 1 μL/mL (Figure 4, EO-RH3). In turn, the concentration of 2 μL/mL caused a gradual decrease in the percentage of apoptotic cells to 14.7% to the level characteristic for the concentration of 0.5 μL/mL. Besides apoptosis, EO-RH4 initiated necrosis at a level of 12.3% and autophagy at a level of 6.3%.

The application of 0.5 μL/mL of EO-RO3 to the T98G culture medium had a considerable effect on cell death, namely, there was a significant 3.5% increase in the number of apoptotic cells (Figure 4, EO-RO3). A further increase in the EO-RO3 concentration also resulted in a statistically significant increase in the level of apoptosis to 28.5%, whereas necrosis was initiated at a level of 1.0% only. In turn, the EO-RO3 concentration of 2 μL/mL caused a decrease in the percentage of apoptotic cells (25.5%), which was not statistically significant. Although apoptosis occurred at this EO-RO3 concentration, necrosis and autophagy were initiated at a level of 26.5% and 2%, respectively.

Our experiments showed a significant 12.7% increase in the number of apoptotic cells upon the addition of EO-RO4 at the concentration of 0.5 μL/mL (Figure 4, EO-RO4). A further increase in the EO-RO4 concentration also resulted in a 32.3% increase in the level of apoptosis, similar to the value of this parameter under the impact of EO-RO3. Besides apoptosis, EO-RO4 initiated necrosis at a low level of 1.8% and autophagy at a level of 1.0%. The EO-RO4 concentration of 2 μL/mL caused a statistically significant decrease in the percentage of apoptotic cells to the level of 25.7%. However, although apoptosis occurred at this EO-RO4 concentration, necrosis and autophagy was initiated at a level of 16.3% and 7.0%, respectively.

Our experiments demonstrated that EO-RH3 and EO-RH4 added to the MOGGCCM culture medium had a considerable effect on induction of cell death (Figure 5, EO-RH3, EO-RH4). A statistically significant increase in the number of apoptotic cells under the influence of the EO-RH3 (18.0%) and EO-RH4 (21.0%) was observed already at the concentration of 1 μL/mL (Figure 4, EO-RH3). The increase in the EO-RH3 and EO-RH4 concentration to 2 μL/mL did not cause significant changes in the level of apoptosis, but contributed to a statistically significant increase in the necrosis level of 17.3% and 19.0%, respectively.

A similar reaction to the EO-RO3 action was observed in the MOGGCCM culture, i.e., a statistically significant increase in the number of apoptotic cells (18.7%) and necrosis (1.3%) at the concentration of 1 μL/mL (Figure 5, EO-RO3). A further increase in the EO-RO3 concentration also resulted in a significant 28.7% increase in the level of apoptosis and a significant 18.7% increase in the level of necrosis. It is worth underlining that the level of apoptosis under the impact of EO-RO3 at the concentration of 2 μL/mL was statistically significantly higher than the values of this parameter obtained in the EO-RH3 and EO-RH4 treatments (Figure 5, EO-RH3, EO-RH4) and simultaneously similar to the values of this parameter obtained under the impact of EO-RO4. Besides apoptosis, EO-RO3 initiated necrosis at a high level of 18.7%.

The application of 0.5 μL/mL of EO-RO4 to the MOGGCCM culture medium had a considerable effect on cell death, namely a significant 6.3% increase in the number of apoptotic cells was observed (Figure 5, EO-RO4). A further increase in the EO-RO4 concentration to 1 μL/mL also resulted in a statistically significant increase in the level of apoptosis to 29.0%, whereas necrosis was initiated at a level of 1.0% only. In turn, the EO-RO4 concentration of 2 μL/mL did not cause a significant change in the percentage of apoptotic cells (27.0%). Although apoptosis occurred at this EO-RO4 concentration, necrosis was initiated at a level of 23.7%. Additionally, it is worth mentioning that none of the EOs studied caused autophagy in the MOGGCCM culture medium.

To sum up, EO-RH3, EO-RH4, and EO-RO4 at the concentration of 1 μL/mL have the most promising influence on apoptosis of the T98G and MOGGCCM cell lines with a low level of necrosis at the same time.

**Figure 6:**
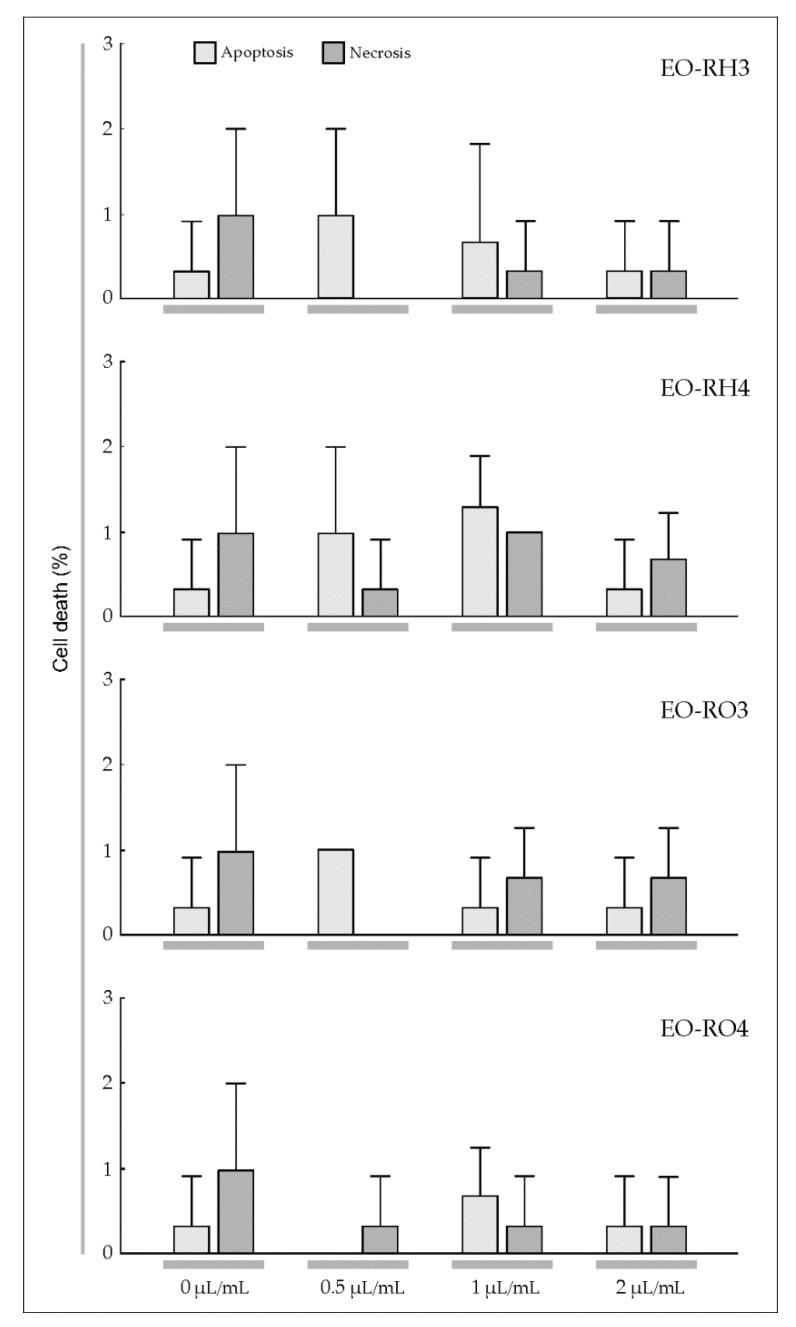
Level of apoptosis and autophagy induction in fibroblasts treated with the essential oil (concentration: 0, 0.5, 1, 2 μL/mL) from the rhizomes and roots of the 3-year-old mountain *Arnica* plants (EO-RH3, EO-RO3) and the rhizomes and roots of the 4-year-old plants (EO-RH4, EO-RO4).

The IC_50_ analysis based on the microscopic observations and colorimetric assay revealed that the MOGGCCM cells were more sensitive to initiation of cell death after the treatment with the essential oils, in comparison to the T98G cell line (Table 4 and Table 5). The analyzed essential oils had no effect on cell death initiation in normal fibroblasts (Figure 6).

## 3. Discussion

The studied EOs were dominated by oxygenated monoterpenes (65.61%, 67.89%, 66.67%, 65,75%) in the EO-RH3, EO-RH4, EO-RO3, and EO-RO4 samples, respectively (Table 1). Phenyls (24.13%, 24.43%, 25.97%, 27.84%) were the second major class of compounds. Another class of constituents was represented by sesquiterpenes (4.67%, 6.11%, 3.03%, 4.04%), monoterpenes (2.05%, 0.87%, 0.95%, 0.49%), and oxygenated sesquiterpenes (0.37%, 0.75%, 0.25%, 0.65%) in the EO-RH3, EO-RH4, EO-RO3, and EO-RO4 samples, respectively.

The chemical composition of the studied EOs from *Arnica* rhizomes and roots is completely different from the EO profile of *Arnica* flower heads collected in different regions of Europe [17,26,41]. In the present study, oxygenated monoterpenes were the most abundant group (65.61%, 67.89%, 66.67%, 65,75%), followed by phenyls (24.13%, 24.43%, 25.97%, 27.84%), sesquiterpenes (4.67%, 6.11%, 3.03%, 4.04%), and monoterpenes (2.05%, 0.87%, 0.95%, 0.49%) in the EO-RH3, EO-RH4, EO-RO3, and EO-RO4 samples, respectively (Table 1). However, EOs obtained from *Arnica* flower heads were dominated by sesquiterpenes (over 60%) [8,9,15,18,21,40]. The main components in the EOs from the analyzed *Arnica* rhizomes and roots were 2,5-dimethoxy-p-cymene (46.47%, 56.42%, 60.31%, 60.23%), 2,6-diisopropylanisole (14.48%, 19.78%, 17.41%, 23.10%), thymol methyl ether (17.79%, 9.18%, 5.31%, 3.87%), p-methoxyheptanophenone (9.65%, 6.19%, 7.02%, 5.07%), and α-isocomene (1.05%, 0.68%, 2.87%, 1.36% in EO-RH3, EO-RH4, EO-RO3, and EO-RO4, respectively. The dominant components of *A. montana* EOs were similar to those demonstrated by Pljevljakušić [17] in rhizomes and roots of two-year-old and three-year-old plants growing on Tara Mountain (subalpine climate) in Serbia. As in our studies, the number of oil constituents in the Serbian population was similar, and the main constituents of rhizome and root oils in two consecutive years were aromatic compounds, i.e., 2,5-dimethoxy-p-cymene (28.9%–30.0% and 37.9%–40.6%, respectively), thymol methyl ether (26.1%–27.1% and 9.6%–10.6%, respectively), p-methoxyheptanophenone (6.1%–8.9% and 7.0%–7.5%, respectively), and 2,6-diisopropylanisole (8.9–10.4% and 12.8–14.1%, respectively). On the other hand, the chemical composition of the studied *A. montana* EOs is very different in terms of the number of EO components from data presented by Weremczuk-Jeżyna [26], where the yields of volatile oils ranged between 0.18% and 1.24%, and the EOs were dominated by 10-isobutyryloxy-8,9-didehydro-thymol isobutyrate and 10-isobutyryloxy-8,9-didehydro-thymol methyl ether. In turn, Danila et al. [41] reported 35 compounds in EO of *A. montana* rhizomes and roots collected from wild populations in the Romanian East-Carpathians, with the following major constituents: 3-t-butyl-1,2-dimethoxybenzene (59.8%–70.5%), thymol (7.5%–9.9%), tetradecane (7.1%–9.8%), non-3-en-2-one (7.4%–8.2%), and thymol methyl ether (5.3%–6.1%). The yields of volatile oils ranged between 2.18% and 3.24%, and there was no significant variation depending on the year of harvest. It is puzzling that the EO profiles of the studied rhizomes and roots are more similar to the EO profile of achenes of *Arnica* originating from the same population [10] than to the EO profile of rhizomes and roots presented by Weremczuk-Jeżyna [26] and Danila [41]. The intraspecific variability of EO components was noted in other plant species from the family Asteraceae, such as *Artemisia absinthium* [6], and *Helichrysum italicum* [7]. Similarly, EOs of *Arnica* flower heads from different European populations have shown their chemical polymorphism [8,9,15,18,21]. However, very interesting is the fact that the proportion of the main EO components from the *Arnica* rhizomes and roots, i.e., 2,5-dimethoxy-p-cymene and thymol methyl ether, is similar to the chemical oil profile in such species as *Ayapana triplinervis* [44,45,46].

As shown by multiyear observations of *Arnica* individuals conducted in field conditions [25], *A. montana* can produce clumping ramets which develop from the short rhizomes of the spreading ramets. In the central part of the four-year-old genets, accumulation of necromass and no production of new ramets were detected, which was a symptom of the onset of genet division. Therefore, the lower EO concentration in the rhizomes of the older *Arnica* plants can be an effect of genet senescence and greater abundance of dead fragments in the raw material. However, the genet division and genet senescence do not explain the lower concentration of EO in the roots of the older *Arnica* plants.

Secondary metabolism is often found to be unique to an organism or is an expression of the individuality of a species [47,48]. Secondary metabolites are produced as a result of organism adaptation to its surrounding environment or are produced to act as a possible defense mechanism against predators and assist in the survival of the organism [48,49]. Biosynthesis of EOs is not only controlled genetically, but is also strongly affected by various biotic and abiotic stresses [50]. Therefore, the EO composition is determined by growth conditions, climate, altitude, soil type, agricultural methods and practices, developmental stage, plant part, and harvesting time [51]. Altitude is one of the abiotic stresses associated with differences in a number of environmental factors such as air temperature, precipitation, light intensity, wind exposure, UV-B radiation, ozone density, and oxidizing air pollutants [52]. A consequence of the altitude differentiation is the variable chemical composition of EOs [53,54]. The presented literature data on the concentration and chemical composition of EO in rhizomes and roots, in comparison with the results of our research, originated from studies carried out in natural habitats and from different field experiments conducted in different regions of Europe, from lowland to mountain regions [17,26,41]. The sources of the plant material differed as well. Therefore, many factors, e.g., edaphic and climatic determinants, have an influence on plant growth and production of primary and secondary metabolites. Probably, climatic conditions are the main determinants of the EO concentration in mountain *Arnica* rhizomes and roots and the chemical composition of EO.

Gliomas are very aggressive brain tumors with very high resistance to chemotherapy [39]; therefore, studies of the effectiveness of different substances in elimination of human glioma cells through apoptosis and autophagy are necessary [35,36,37,38]. Jakubowicz-Gil et al. [35,36,37,38] made an attempt to use single natural chemicals, such as quercetin, or combinations with temozolomide [35,36,37] and sorafenib [38], which were shown as potent apoptosis inducers in T98G and MOGGCCM cells. Conventional cancer therapies cause serious side effects [28]. However, many alternative treatments and therapies based on plants have been explored in the recent years, which is especially important for patients that do not tolerate extreme side effects [46]. Plants have been analyzed to identify their anticancer properties and characterized chemically to reveal the presence of many bioactive compounds [29,30]. The effect of EOs with a specific chemical composition has been reported to vary depending on the features of particular cancer types. Ribeiro et al. [55] investigated the cytotoxic potential of *L. gracilis* EOs against tumor cell lines. The results showed that the oils inhibited human melanoma (MDA-MB-435) and colon carcinoma (HCT-8) cell lines. Lima et al. [56] indicated that the cytotoxicity of EOs from *L. lucidus* against liver carcinoma and breast cancer cell lines was stronger than against other cell lines. da Silva et al. [57] showed varied cytotoxic activity of *Piper aleyreanum* oils on colon and melanoma cell lines. Similarly, Yu et al. [58] demonstrated various levels of cytotoxic activity of *P. klotzschianum* oils inhibiting human hepatocellular carcinoma, human promyelocytic leukemia, and murine melanoma cell lines. A broad cytotoxicity spectrum was indicated in the case of essential oil of *P. cernuum* against human melanoma, human cervical tumor, and human glioblastoma cells [59,60]. Considering the anticancer activity of the EOs described in the present study and the level of apoptosis caused by EOs extracted from the underground parts of *A. montana* (Figure 4 and Figure 5), this plant species can be included in the medicinal plant group with high anticancer potential.

Apoptosis is one of the main mechanisms by which chemotherapeutic agents induce cell death in cancer cells [61]. The increase in the EO-RH3, EO-RH4, EO-RO3, EO-RO4 concentration to 1 μL/mL resulted in an increase in the level of apoptosis to 18.0%, 21.0%, 28.7%, and 29% in the MOGGCCM cell line, respectively (Figure 4). Similar results were obtained in the case of the T98G cell line, namely, the increase in the EO-RH3, EO-RH4, EO-RO3, EO-RO4 concentration to 1 μL/mL resulted in an increase in the level of apoptosis to 32.3%, 19.7%, 28.5%, and 32.3%, respectively (Figure 5), which suggests that the essential oil is a promising natural anticancer product. In the world literature, there is another report of the effect of *A. montana* essential oil on the induction of apoptosis of glioma T98G and MOGGCCM cell lines [10]; hence, this species can be regarded as an important source of anticancer secondary metabolites. This is an important finding, especially in the light of recent investigations showing that gliomas naturally resist apoptosis [62].

Exploration of natural plant products as an option to find new chemicals as anticancer agents is one of the fastest growing areas of research, and the available studies indicate EOs from various plant species and their constituents as anticancer agents [63]. A recent study has shown that EOs can be a potential alternative for glioblastoma treatment [64,65,66]. Quassinti et al. [65] showed that EO of *Hypericum hircinum* had antiproliferative activity on human glioblastoma tumor cells T98G. Detoni et al. [64] demonstrated that EO of *Zanthoxylum tinguassuiba* could be a potential alternative for gliobastoma treatment. As reported by Bayala et al. [66], essential oil of *Ageratum conyzoides* shows the highest antitumor activity on SF-763 cells, while the SF-767 glioblastoma cell line was the most sensitive to EOs of *Ocimum basilicum* and *Lippia multiflora*. In the literature, there are examples of research on the effects of EOs or their main ingredients on glioma cells. Thymol, i.e., the main component of many aromatic and medicinal plants has been shown to have a stimulating effect on apoptosis and an inhibitory effect on cell growth in DBTRG-05MG human glioblastoma [63]. α-Bisabolol, which is a natural compound strongly inducing apoptosis in glioma cells [2], or β-Elemene, i.e., a natural plant drug obtained from *Curcuma wenyujin* inducing apoptosis in glioblastoma cells, exert promising anticancer effects against a broad spectrum of tumors [32,67]. Another example of molecules characterized by an anticancer effect on human glioblastoma cell line U87MG is aloe emodin, i.e., an anthraquinone compound present in the leaves of *Aloe arborescens*, and hispolon, which is a polyphenolic compound isolated from *Phellinus linteus* [68]. The study presented in this paper is the first attempt to search for phytochemicals contained in *Arnica* rhizomes and roots characterized by antiglioma activity. Our earlier study showed anticancer activity of EOs from mountain *Arnica* achenes on the T98G and MOGGCCM cell lines [10]. Puzzling is fact that the EOs from achenes have a similar chemical composition to that from rhizomes and roots, with 2.5-dimethoxy-p-cymene, thymol methyl ether, and 2,6-diisopropylanisole as the main ingredients.

The components of the EOs present in the analyzed *Arnica* rhizomes and roots determine the biological activity of formulations derived from these plant raw materials. 2,5-dimethoxy-p-cymene, thymol methyl ether, p-methoxyheptanophenone, and 2,6-diisopropylanisole are the main components of the EOs in the underground parts of mountain *Arnica*. In view of continuation of the study, it is highly promising that the medicinal plant *A. triplinervis* from the family Asteraceae has recently been identified for the first time as a new source of antiviral phytocompounds targeting the entry of viral pathogens to cells, with 2.5-dimethoxy-p-cymene as the main component of EO extracted from leaves [46]. The study on the use of 2.5-dimethoxy-p-cymene has demonstrated that this molecule isolated from *A. triplinervis* EO is a potent inhibitor of ZIKV infection in human cells. Moreover, assessment of the biosafety of 2.5-dimethoxy-p-cymene extracted from *A. triplinervis* has shown that injection of this molecule in zebrafish does not lead to any signs of stress and does not affect fish survival, demonstrating the absence of acute toxicity of this compound [46]. However, different results were obtained by Unnikrishnan et al. [45]. Namely, the essential oils from *Eupatorium triplinerve* stems and leaves were found to have a significant antimicrobial effect, compared to that of 2.5-dimethoxy-p-cymene. Hence, the researchers recapitulated that the antimicrobial effect of the EO could be attributed to its minor components [45]. In turn, thymol methyl ether is the major constituent of EO of *Crithmum maritimum* [69], exhibits considerable activity mostly against Gram+ bacteria [70], and is one of the main ingredients of EO in the aerial parts of *Baccharis grisebachii* characterized by antimicrobial properties [71]. This molecule is also one of the major constituents of EO isolated from aerial parts of several *Thymus* species [72,73]. EO of *T. numidicus* is characterized by insecticidal properties [73], whereas EO isolated from aerial parts of *T. fontanesii* showed strong in vitro growth inhibition activity against Gram– bacteria and antifungal activity [72].

Plant-derived compounds have a high impact as therapeutic agents, both alone and in combination with conventional drugs [74]. The literature shows many examples of the potential synergistic, additive, or even antagonistic biological effects of combinations of individual EO compounds applied at different concentrations [74,75,76]. Generally, the biological activity of an EO is related to its chemical composition and the major functional groups of compounds, and the components at high concentrations play a major role in the biological effect of EOs. However, less abundant compounds may also be important, as various molecules can act synergistically with the major compounds [77]. Our results showed no negative effect of EOs on the normal cell line (Table 2). This is very important in the context of our further studies of the anticancer and antiglioma activity of EORH and EORO. Therefore, in the continuation of the studies of the antiglioma activity, not only the dominant EO ingredient 2.5-dimethoxy-p-cymene but also less abundant compounds (alone and in combination) will be taken into account. The mechanisms of action of these main bioactive phytochemical compounds contained in the studied EO (2,5-dimethoxy-p-cymene, thymol methyl ether, p-methoxyheptanophenone, 2,6-diisopropylanisole) are unknown, similarly as the metabolic pathways responsible for biosynthesis of these molecules.

It is obvious that experiment conducted on pure secondary metabolites will bring more valuable and precise information about anticancer activity of studied extracts. Therefore, more detailed experiments toward estimating the precise molecular mechanism leading to cell death upon EOs and purified compounds will be continued in the near future. Given our previous experience in studying isolated flavonoids (quercetin) and coumarins (osthole, imperatorin), the research will be continued in accordance to anti-migratory potential, the level of typical for cell death marker proteins and related genes expression. We will also try to combine isolated compounds with cytostatic drugs used in anticancer therapy to assess their usefulness in combination therapy. Because such experiments were not undertaken yet, we hope to receive some new and valuable information with practical potential. Therefore, further studies on the anticancer effect of *Arnica* EOs and the main EO components (individually and in combination) against deadly gliomas will be an attempt at elucidation of the molecular anticancer mechanisms of this interesting constituent of *A. montana* EO.

## 4. Materials and Methods

### 4.1. Collection of Raw Material

Mountain *Arnica montana* subsp. *montana* plants were harvested at the end of the third and fourth vegetation periods from two experimental fields (3-year-old and 4-year-old plantation) at the University of Life Sciences in Lublin, eastern Poland. Standard fertilization was used: nitrogen (40.0 kg ha^−1^), phosphorus (24.0 kg ha^−1^), and potassium (66.4 kg ha^−1^) were applied every year. After harvesting randomly selected 3-year-old and 4-year-old *Arnica* plants, their underground parts were carefully washed with tap water and finally with deionized water. After washing, the rhizomes and roots were separated from underground organs and subsequently air-dried and crushed.

### 4.2. Qualitative and Quantitative Analysis of Essential Oil

#### 4.2.1. Assay of the Essential Oil Content

Twenty grams of crushed rhizomes and roots (mean particle size—0.5 mm) of mountain *Arnica* were submitted to water-distillation in a Deryng apparatus with 500 mL water for 3 h according to the Polish Pharmacopoeia VI [78]. The method of indirect distillation was applied. The essential oils were collected over water, separated, dried over anhydrous sodium sulphate, and stored in the dark at 4 °C prior to GC–MS analysis. The analysis was carried out in four repetitions. The moisture content of the mountain *Arnica* rhizomes and roots (9.62%) was determined by heating 10 g of the raw material at a temperature of 105 °C in an air oven until three consecutive constant weights were recorded using an electronic balance measuring with an accuracy of 0.001 g. The essential oil content was calculated to absolute dry weight.

#### 4.2.2. GC-MS Analysis

The chromatographic analysis was performed according to procedures described previously [9,21]. The analysis was performed in triplicate. The essential oils were analyzed using a Varian 4000 GC–MS/MS system (Varian, Palo Alto, California, CA, USA). The compounds were separated on a 30 m × 0.25 mm × 0.25 μm VF–5 ms column (Varian, Palo Alto, California, CA, USA). The column temperature was increased from 50 to 250 °C at a rate of 4 °C/min; injector temperature 250 °C; split ratio 1:50; injection volume 5 μL. The MS parameters were as follows: EI mode, with ionization voltage 70 eV, ion source temperature, 200 °C; scan range, 40–870 Da.

#### 4.2.3. Qualitative and Quantitative Analysis

The qualitative analysis was carried out on the basis of MS spectra, which were compared with the spectra from the NIST library [79] and with data available in the literature [43,80]. The identity of the compounds was confirmed by their retention indices [42] taken from the literature [43,80] and our data for standards described previously [9,21]. The quantitative analysis was performed with the internal standard addition method (alkanes C_12_ and C_19_) according to procedures described previously [81].

### 4.3. Glioma Cells and Culture

#### 4.3.1. Cells and Culture Conditions

Human glioblastoma multiforme cells (T98G, European Collection of Cell Cultures) and human anaplastic astrocytoma cells (MOGGCCM, European Collection of Cell Cultures) were grown in a 3:1 mixture of Dulbecco’s Modified Eagle Medium (DMEM) and Ham’s nutrient mixture F-12 (Sigma, St. Louis, MO, USA) supplemented with 10% fetal bovine serum (Sigma), penicillin (100 units/mL) (Sigma), and streptomycin (100 μg/mL) (Sigma, St. Louis, MO, USA). The cultures were kept at 37 °C in a humidified atmosphere of 95% air and 5% CO_2_. Primary cultures of human skin fibroblast were prepared according to a method described previously [82].

#### 4.3.2. Detection of Apoptosis, Necrosis, and Autophagy

Apoptosis, autophagy, and necrosis in control (0 μL/mL) and EO treated cells (0.5 μL/mL (0.415 mg/mL), 1 μL/mL (0.830 mg/mL), 2 μL/mL (1.660 mg/mL)) were identified microscopically after staining with fluorochromes Hoechst 33342 (Sigma), acridine orange (Sigma, St. Louis, MO, USA), and propidium iodide (Sigma, St. Louis, MO, USA) respectively, as described previously [59,60,61]. A fluorescence microscope (Nikon E-800, Tokyo, Japan) was used for morphological analysis of dead cells. At least 1000 cells in randomly selected microscopic fields were counted under the microscope. Each experiment was repeated three times with each 1000 cells. In addition, 50% inhibitory concentrations (IC_50_ values, μL/mL) were determined for all the tested extracts using GraphPad Prism version 7 (GraphPad Software, San Diego, CA, USA).

#### 4.3.3. Neutral Red Staining

To confirm the ID50 values (μL/mL) obtained from the microscopic observation, a colorimetric assay with the Neutral Red Assay Kit—Cell Viability/Cytotoxicity (ab234039, Abcam) was used. The principle of this assay is based on the detection of viable cells via the uptake of the neutral red dye. Viable cells can take up neutral red via active transport, whereas non-viable cells cannot take up this chromophore. The neutral red uptake assay was performed according to manufacturer’s protocol. OD 540 nm was measured with a BioTek 800 TS microplate reader. The experiment was performed in triplicate.

### 4.4. Statistical Analysis

One-way and multi-way analysis of variance (ANOVA) and subsequent Tukey’s tests were used. The differences were considered significant at *p* < 0.05. The statistical analyses were carried out using the Statistica 6.0 software (Stat. Soft, Inc., Kraków, Polska). Principal component analysis (PCA) was applied to explain the relationships between oil components and to show variability factors. Prior to the PCA, the data on all 37 components of EO were centered. The analyses were carried out using the statistical package (MVSP) program version 3.1. [83].

## 5. Conclusions

Among the 37 components of the essential oil of *A. montana*, 2,5-dimethoxy-p-cymene (46.47%, 56.42%, 60.31%, 60.23%), 2,6-diisopropylanisole (14.48%, 19.78%, 17.41%, 23.10%), thymol methyl ether (17.79%, 9.18%, 5.31%, 3.87%), p-methoxyheptanophenone (9.65%, 6.19%, 7.02%, 5.07%), and α-isocomene (1.05%, 0.68%, 2.87%, 1.36%), were detected in the rhizomes and roots of the 3-year-old plants and in the rhizomes and roots of the 4-year-old plants. Their quality and chemical composition are different from those of essential oils from flower heads, achenes, and rhizomes and roots of *Arnica* populations in different regions of Europe. Essential oils exert an anticancer effect by induction of anaplastic astrocytoma and glioblastoma multiforme cell death. Given the activity expressed by induction of apoptosis at a level of 28.5%–32.3%, the substance can be used in subsequent studies focused on the development of glioma therapy in the future. The plant part (rhizome, root) and plant age can be determinants of the concentration and essential oil composition and, consequently, their biological activity. The knowledge and information obtained in this study indicate a need for further research on the anticancer effect of EO components (individually and in combination) on the T98G and MOGGCCM cell lines and elucidation of the molecular anticancer mechanisms of this interesting constituent of *A. montana* EOs constituting a potential source of natural anticancer compounds.

## Figures and Tables

**Table 1:** Composition of essential oils from *A. montana* rhizomes and roots of the 3-year-old plants (RH3, RO3) and in the rhizomes and roots of the 4-year-old plants (RH4, RO4). The values designated by the different letters (essential oil content) are significantly different (*p* = 0.05). (Tukey-test, *p* < 0.05).

			**RH3**	**RH4**	**RO3**	**RO4**
***A. Montana*** **Rhizomes and Roots**			Essential Oil Content [% v/w] ± SD			
			0.664 ^b^ ± 0.026	0.518 ^a^ ± 0.029	0.844 ^c^ ± 0.023	0.657 ^a^ ± 0.023
Compound	RI	RI_Lit_	Essential Oil Composition [%] ± SD			
Cumene	928	931 ^a^	0.14 ± 0.015	0.20 ± 0.002	0.08 ± 0.001	0.09 ± 0.002
α-pinene	935	939 ^a^	0.03 ± 0.002	0.02 ± 0.002	0.01 ± 0.000	0.01 ± 0.001
Camphene	950	954 ^a^	0.13 ± 0.010	0.09 ± 0.001	0.06 ± 0.001	0.05 ± 0.002
β-pinene	976	979 ^a^	0.00 ± 0.004	–	–	–
δ-2-carene	998	1002 ^a^	0.03 ± 0.003	0.01 ± 0.008	0.01 ± 0.001	0.01 ± 0.001
α-phellandrene	1000	1003 ^a^	0.62 ± 0.034	0.24 ± 0.006	0.24 ± 0.003	0.10 ± 0.002
α-terpinene	1014	1017 ^a^	0.01 ± 0.005	–	–	–
p-cymene	1022	1025 ^a^	0.73 ± 0.040	0.19 ± 0.004	0.27 ± 0.003	0.09 ± 0.000
Sylvestrene	1028	1031 ^a^	0.30 ± 0.011	0.17 ± 0.002	0.17 ± 0.002	0.12 ± 0.001
Terpinolene	1086	1089 ^a^	0.07 ± 0.005	0.04 ± 0.001	0.03 ± 0.001	0.02 ± 0.000
Z-p-menth-2-en-1-ol	1118	1122 ^a^	0.04 ± 0.004	0.02 ± 0.002	0.03 ± 0.000	0.03 ± 0.002
E-p-menth-2-en-1-ol	1139	1141 ^a^	0.03 ± 0.017	0.01 ± 0.008	0.02 ± 0.001	0.02 ± 0.000
γ-terpineol	1197	1199 ^a^	0.03 ± 0.030	0.05 ± 0.002	0.07 ± 0.001	0.10 ± 0.002
E-piperitol	1215	1208 ^a^	0.01 ± 0.007	0.01 ± 0.000	0.01 ± 0.007	0.01 ± 0.001
Thymol methyl ether	1234	1235 ^a^	17.79 ± 0.284	9.18 ± 0.103	5.31 ± 0.054	3.87 ± 0.012
Carvacrol methyl ether	1243	1245 ^a^	0.41 ± 0.009	0.31 ± 0.005	0.31 ± 0.002	0.31 ± 0.002
Isobornyl acetate	1285	1286 ^a^	0.18 ± 0.009	1.04 ± 1.400	0.18 ± 0.002	0.18 ± 0.002
Thymol	1288	1290 ^a^	0.37 ± 0.032	0.55 ± 0.018	0.26 ± 0.003	0.49 ± 0.002
Silphiperfol-5-ene	1328	1329 ^a^	0.14 ± 0.002	0.13 ± 0.005	0.27 ± 0.002	0.27 ± 0.002
Presilphiperphol-7-ene	1336	1337 ^a^	0.01 ± 0.008	–	0.03 ± 0.006	0.02 ± 0.001
7-epi-silphiperfol-5-ene	1345	1348 ^a^	0.40 ± 0.007	0.27 ± 0.036	0.78 ± 0.018	0.55 ± 0.002
Silphiperfol-6-ene	1377	1379 ^a^	0.01 ± 0.001	0.02 ± 0.005	0.03 ± 0.001	0.04 ± 0.001
β-maliene	1381	1382 ^a^	0.11 ± 0.003	0.06 ± 0.004	0.23 ± 0.003	0.11 ± 0.001
α-isocomene	1387	1388 ^a^	1.05 ± 0.009	0.68 ± 0.006	2.87 ± 0.036	1.36 ± 0.006
β-isocomene	1404	1407 ^a^	0.04 ± 0.001	0.04 ± 0.001	0.03 ± 0.001	0.03 ± 0.001
E-caryophyllene	1416	1419 ^a^	1.02 ± 0.025	0.55 ± 0.003	0.27 ± 0.001	0.17 ± 0.005
2.5-dimethoxy-p-cymene	1424	1427 ^a^	46.47 ± 0.291	56.42 ± 0.068	60.31 ± 0.200	60.23 ± 0.102
E-α-bergamotene	1431	1435 ^a^	0.62 ± 0.004	0.45 ± 0.014	0.59 ± 0.007	0.40 ± 0.004
2.6-diisopropylanisole	1435	1438 ^b^	14.48 ± 0.436	19.78 ± 0.074	17.41 ± 0.043	23.10 ± 0.168
Z-β-farnesene	1440	1443 ^a^	0.09 ± 0.003	0.05 ± 0.005	0.09 ± 0.002	0.05 ± 0.002
α-humulene	1452	1455 ^a^	0.10 ± 0.002	0.07 ± 0.003	0.08 ± 0.002	0.06 ± 0.000
E-β-farnesene	1454	1457 ^a^	0.16 ± 0.002	0.12 ± 0.011	0.07 ± 0.001	0.05 ± 0.000
p-methoxyheptanophenone	1480	1476 ^b^	9.65 ± 0.015	6.19 ± 0.047	7.02 ± 0.052	5.07 ± 0.074
Germacrene D	1483	1485 ^a^	0.37 ± 0.005	0.25 ± 0.004	0.03 ± 0.001	0.37 ± 0.020
Isobornyl isovalerate	1520	1523 ^a^	0.27 ± 0.012	0.28 ± 0.000	0.17 ± 0.002	0.18 ± 0.003
β-sesquiphellandrene	1521	1523 ^a^	0.54 ± 0.004	0.34 ± 0.003	0.72 ± 0.003	0.58 ± 0.005
Lippifoli-1(6)-en-5-one	1550	1553 ^a^	0.37 ± 0.004	0.25 ± 0.006	0.75 ± 0.015	0.65 ± 0.011
Monoterpenes			2.05	0.95	0.87	0.49
Oxygenated monoterpenes			65.61	67.89	66.67	65.75
Sesquiterpenes			4.67	3.03	6.11	4.04
Oxygenated sesquiterpenes			0.37	0.25	0.75	0.65
Phenyls			24.13	25.97	24.43	27.84
Sum of Identified (%)			96.82	98.09	98.84	98.78

RI—retention indices (from temperature-programming using the definition proposed by Van Den Dool and Kratz [42]); RI_Lit_—retention indices taken from literature [43] ^a^, [17] ^b.^

**Table 2:** Results of PCA based on EO components. (**a**) Eigenvalues and variance (%) explained by the first two PCA axes; (**b**) loading components for each variable associated with the two axes.

Chemical Variables	Axis 1	Axis 2
(**a**)		
Eigenvalues	76.51	5.07
Percentage	92.77	6.15
Cumulative percentage	92.77	98.93
(**b**)		
2,5-dimethoxy-p-cymene	0.668	−0.309
thymol methyl ether	−0.645	0.137
2,6-diisopropylanisole	0.313	0.822
p-methoxyheptanophenone	−0.187	−0.278
α-isocomene	0.040	−0.333

**Table 3:** Effect of the main factors and their interactions on the level of apoptosis, necrosis, and autophagy. Result of multi-way analysis of variance (ANOVA). T98G—glioblastoma multiforme cell line, MOGGCCM—anaplastic astrocytoma cell line.

Cell Line		T98G		MOGGCCM	
Factor	Apoptosis	Necrosis	Autophagy	Apoptosis	Necrosis
Part plant (R)	*F* = 1.0,	F = 0.2,	F = 0.1,	F = 56.2,	F = 3.0,
	*p* = 0.313	*p* = 0.661	*p* = 0.876	*p* < 0.001	*p* = 0.095
Plant age (A)	F = 4.7,	F = 8.0,	F = 7.7,	F = 9.9,	F = 2.1,
	*p* = 0.037	*p* = 0.008	*p* = 0.009	*p* = 0.004	*p* = 0.160
EO concentration (C)	F = 1895.3,	F = 279.2,	F = 66.1,	F = 6747.6,	F = 242.2,
	*p* < 0.001	*p* < 0.001	*p* < 0.001	*p* < 0.001	*p* < 0.001
R × A	F = 48.2,	F = 1.4,	F = 1.1,	F = 12.3,	F = 1.7,
	*p* < 0.001	*p* = 0.250	*p* = 0.294	*p* = 0.001	*p* = 0.201
R × C	F = 50.7,	F = 0.1,	F = 4.2,	F = 13.7,	F = 2.2,
	*p* < 0.001	*p* = 0.976	*p* = 0.013	*p* < 0.001	*p* = 0.111
A × C	F = 12.0,	F = 4.7,	F = 9.9,	F = 16.5,	F = 0.2,
	*p* < 0.001	*p* < 0.008	*p* < 0.001	*p* < 0.001	*p* = 0.871
R × A × C	F = 21.2,	F = 0.5,	F = 6.1,	F = 3.1,	F = 1.1,
	*p* < 0.001	*p* = 0.710	*p* = 0.002	*p* = 0.042	*p* = 0.348

**Table 4:** Fifty percent inhibitory concentrations (IC_50_ value, μL/mL) for the essential oil from the rhizomes and roots of the 3-year-old mountain *Arnica* plants (EO-RH3, EO-RO3) and the rhizomes and roots of the 4-year-old plants (EO-RH4, EO-RO4) after 24 h long incubation.

Cell Line	EO-RH3	EO-RH4	EO-RO3	EO-RO4
T98G	1.9	3.0	2.0	2.0
MOGGCCM	2.6	2.7	2.1	1.9

**Table 5:** Fifty percent inhibitory concentrations (IC_50_ value, μL/mL) for the essential oil from mountain *Arnica* rhizomes and roots of the 3-year-old plants (EO-RH3, EO-RO3) and in the rhizomes and roots of the 4-year-old plants (EO-RH4, EO-RO4) after 24 h long incubation and staining with neutral red.

Cell Line	EO-RH3	EO-RH4	EO-RO3	EO-RO4
T98G	2.3	2.6	2.1	2.0
MOGGCCM	2.1	2.0	2.1	1.9
